# Icephobicity of Hierarchically Rough Aluminum Surfaces Sequentially Coated with Fluoroalkyl and PDMS Alkoxysilanes

**DOI:** 10.3390/polym15040932

**Published:** 2023-02-13

**Authors:** Tien N. H. Lo, Ha Soo Hwang, In Park

**Affiliations:** 1 Research Institute of Clean Manufacturing System, Korea Institute of Industrial Technology (KITECH), 89 Yangdaegiro-gil, Ipjang-myeon, Cheonan-si 31056, Chungcheongnam-do, Republic of Korea; 2 R&D Center, OomphChem Inc., 1223-24 Cheonan-daero, Seobuk-gu, Cheonan-si 31080, Chungcheongnam-do, Republic of Korea; 3 KITECH School, University of Science and Technology (UST), 176 Gajeong-dong, Yuseong-gu, Daejeon-si 34113, Chungcheongnam-do, Republic of Korea

**Keywords:** superhydrophobic, icephobicity, anti-icing, ice adhesion, delayed freezing

## Abstract

Superhydrophobic surfaces fabricated by grafting 1H,1H,2H,2H-heptadecafluorodecyl trimethoxysilane (FD-TMS) and polydimethylsiloxane triethoxysilane (PDMS-TES) onto a nano-micro hierarchical aluminum (Al) surface are considered to possess substantial anti-icing functionality, with delayed freezing and low ice-adhesion strength (IAS). Verifying the impacts of PDMS and the synergism of PDMS and FD on the anti-icing performance is the goal of this study. Roughness, one of the prerequisites for superhydrophobicity, was obtained by etching Al substrates in aqueous HCl, followed by immersion in boiling water. FD-TMS and PDMS-TES were then coated on the rough Al substrates layer by layer; a congener coated with a single layer was also prepared for comparison. The FD-PDMS_1.92_ (1.92 wt.%) coating, in which FD-TMS and PDMS-TES were used as primary and secondary coating materials, respectively, exhibited superior icephobicity, with the lowest IAS of 28 kPa under extremely condensing weather conditions (−20 °C and 70% relative humidity, RH) and the longest freezing delay time of 230 min (at −18 °C). These features are attributed to the incorporation of a dense coating layer with a low-surface-tension FD and the high mobility of PDMS, which lowered the contact area and interaction between the ice and substrate. The substrate coated with FD-PDMS_1.92_ exhibited improved durability with an IAS of 63 kPa after 40 icing/melting cycles, which is far less than that achieved with the FD single-layer coating.

## 1. Introduction

In the winter, water (typically rain) droplets become frozen and form ice. Tragic accidents are caused by ice on the surface of roads and aircrafts [1]. Power transmission lines, towers, and wind turbine blades may become distorted or collapse due to the heavy weight of accumulating ice. In addition to icing, frost produced by the nucleation and condensation of water vapor causes problems such as poor heat transfer efficiency in frosted heat exchangers. Therefore, there is an urgent need to find a solution to the problems caused by ice formation on solid substrates. Current industrial strategies for anti-icing (preventing or delaying icing or reducing ice adhesion) and melting (removal of ice from a surface) primarily involve chemical melting fluids [2], mechanical removal [3], and electrothermal treatment [4,5]. However, these methods are associated with environmental contamination, high energy costs, and long treatment times. Therefore, as a more appealing and universal approach, passive coating on solid surfaces is used to minimize ice adhesion, enabling facile removal of the accumulated ice by natural forces such as wind, gravity, or vibration.

Because ice adhesion is directly associated with the interaction between the ice and substrate, a good icephobic coating should have a low surface tension [6]. In order to weaken the ice–substrate interaction, it is necessary to lower the surface tension and reduce the actual ice–solid contact area by introducing hierarchical micro-nano roughness on the substrate. Based on this consideration, current studies on icephobic materials have mainly focused on superhydrophobic surfaces, which combine the synergistic impact of surface roughness and low surface tension [7,8,9]. Fluorochemicals, silicones, and silane coupling agents are low-surface-tension compounds that are frequently utilized as coating materials [10,11,12]. Fluoroalkylsilanes are regarded as the most hydrophobic and icephobic materials owing to their extremely low surface tension [13,14,15]. The formation of a dense and well-organized film on substrates using silanes is another crucial approach for achieving hydrophobicity and ice repellency [16].

Although polysiloxanes (e.g., PDMS) have a slightly higher surface free energy than fluorinated materials, their lower interaction energy with ice or water molecules and their high flexibility make them prospective candidates for anti-icing materials [17]. Polysiloxanes are also more beneficial and suitable for practical use than fluorinated materials because they are more environmentally friendly and cost-effective. A large diversity of polysiloxane icephobic surfaces with extremely low ice-adhesion strength (IAS) has been reported [18,19]. Despite the limited number of studies in which PDMS coatings are used on rough Al plates, silicon rubber or cross-linked PDMS with high molecular weight partially filled cavities in the superhydrophobic structure has been used to reduce the surface roughness [20]. Polysiloxanes should have functional groups that form stable covalent bonds with surfaces. To improve the anti-icing properties, the molecular weight of polysiloxanes should be low enough to prevent cavities from filling on the rough surface of superhydrophobic Al substrates.

In our prior research, we demonstrated the significant anti-icing properties of hierarchically rough Al plates, achieved by coating with FD-silane and using a suitable PDMS-silane ratio [21]. Due to the low interaction energy of highly flexible PDMS with water and the low surface tension of the FD, the Al substrate coated with both PDMS and FD showed a lower IAS (25.3 kPa at −20 °C, 75% RH) and longer ice nucleation time (204.5 min at −18 ℃) compared to the Al substrate coated with only FD (141.3 kPa and 71.5 min). After 100 cycles of icing/melting, the IAS was 47.2 kPa, and exceptional robustness was also achieved because an adequate ratio of chemically reactive PDMS-silane to the substrate was used. Although we found that flexible PDMS and low-surface-tension FD worked together to achieve icephobicity, the mechanism at the interface is still mostly unknown.

In this study, superhydrophobic surfaces with ultralow ice-adhesion strength (<50 kPa) and high durability are fabricated by sequentially coating hierarchically rough Al substrates with FD and PDMS to clarify the influence of the coating order of PDMS and FD on the icephobicity by employing four sequential FD/PDMS coatings. The wettability, icephobicity, and durability are studied based on the contact angle (CA), sliding angle (SA), IAS, and freezing delay time. The wettability and icephobicity of these surfaces differ tremendously. These differences are discussed in terms of the surface properties, such as the surface tension, surface coverage, and the ice–substrate interaction effect. For the rough Al substrate coated in sequence with FD and PDMS, the outer PDMS layer acts as a lubricant that is covalently immobilized on the substrate, resulting in enhanced durability. Otherwise, the poor surface coverage or high interaction energy of the coated Al surface with water increases the IAS and promotes the ice formation of water on the surface. The findings of this study offer a new strategy for designing solid surfaces with exceedingly high icephobicity.

## 2. Materials and Methods

### 2.1. Materials

Al alloy plates (Al 1100) were obtained and cut into square shapes with dimensions of 50 mm × 50 mm × 0.8 mm. Sooyang Chemtec provided 1H,1H,2H,2H-heptadecafluorodecyl trimethoxysilane (FD-TMS) (Eumseong-gun, Republic of Korea). Gelest supplied monotriethoxysilylethyl-terminated polydimethylsiloxane (16–24 cst; PDMS-TES). Toluene (99.5%), acetone (99.5%), ethanol (99.5%), and concentrated HCl solution were provided by Samchun Chemicals (Yeosu-si, Republic of Korea). None of the compounds were further purified before use.

### 2.2. Sample Preparation

The following procedure was used to coat FD and PDMS on the hierarchical micro/nanostructured Al surface via a layer-by-layer approach. To produce hierarchical roughness, the Al substrates were first cleaned with ethanol and acetone and then etched in an aqueous HCl solution (2.5 M) at 25 °C for 10 min. The etched Al plate was then dipped in boiling water for 30 min to generate a hierarchical micro/nanostructured surface, as explained in our previous study [7]. Subsequently, the sample was dipped in a 1 wt.% toluene solution of FD-TMS or PDMS-TES with different depositional sequences. Four types of superhydrophobic surfaces with different sequential coating layers were prepared.

FD_100_: The micro/nanostructured Al plates were coated with FD-TMS by immersion in an FD-TMS solution (1 wt.% in toluene) at room temperature for 2 h and then dried at 150 °C for 24 h.

PDMS_100_: The micro/nanostructure Al plates were coated with PDMS-TES by immersion in a PDMS-TES solution (1 wt.% in toluene) at 60 °C for 2 h, followed by drying at 150 °C for 24 h.

FD-PDMS_1.92_: The hierarchically rough Al plate was first modified with the FD coating using the same coating procedure as used for FD_100_. After drying for 15 min at 60 °C, the plate was coated with PDMS using the same coating procedure as applied to PDMS_100_, as mentioned above. Subsequently, the samples were dried at 150 °C for 24 h.

PDMS_4.0_-FD: The PDMS-FD-coated Al substrate was prepared in the similar way as the FD-PDMS sample with a reversed procedure: primary coating using PDMS-TES and secondary coating with FD-TMS.

### 2.3. Characterizations

^1^H NMR spectra were recorded on a Brucker 300 MHz spectrometer (Brucker corporation) to calculate the weight ratio of PDMS-Si tethered on the Al substrate by scraping the coated Al plate with a single edge razor blade in deuterochloroform. The characteristic peaks, –CF_2_–CF_2_–C*H*_2_– and –O–Si–(C*H*_3_)_2_– for FD-Si and PDMS-Si, respectively, were integrated to calculate the weight ratios of PDMS-Si or FD, and they were denoted x in FD-PDMSx or PDMS-FDx. The surface topologies were observed by a field emission scanning electron microscope (FE-SEM; JSM-6701F, JEOL, Tokyo, Japan) with a beam intensity of 5 kV. The CA was measured with a 5 μL droplet of water or hexadecane at 25 °C using a Smart Drop CA system (Femtobiomed, Seongnam-si, Republic of Korea) according to the sessile drop method. The slant angles were recorded and used to calculate SA when the droplet rolled off the surface. A Kα X-ray photoelectron spectrometer (XPS; Thermo Scientific, Waltham, MA, USA) was used to examine the atomic composition of the surfaces using a monochromatic Al Kα X-ray source with photon energy of 1486.6 eV. Electrochemical measurements were carried out using a three-electrode system (VSP 300; Bio-Logic SAS, Seyssinet-Pariset, France) in a 3.5 wt.% NaCl solution at 25 °C. A saturated calomel electrode (SCE, reference electrode) and a graphite rod counter electrode were used in a three-electrode cell for the measurements. The work electrode was a sample with an exposed area of 1 by 1 cm. All samples were dipped in 3.5 wt.% NaCl solution for 60 min prior to electrochemical measurements to acquire a consistent open-circuit potential (OCP). Electrochemical impedance spectroscopy (EIS) experiments were subsequently carried out at an amplitude of 20 mV in the frequency range of 0.01–100,000 Hz.

### 2.4. Evaluation of Anti-Icing Properties

**Figure 1:**
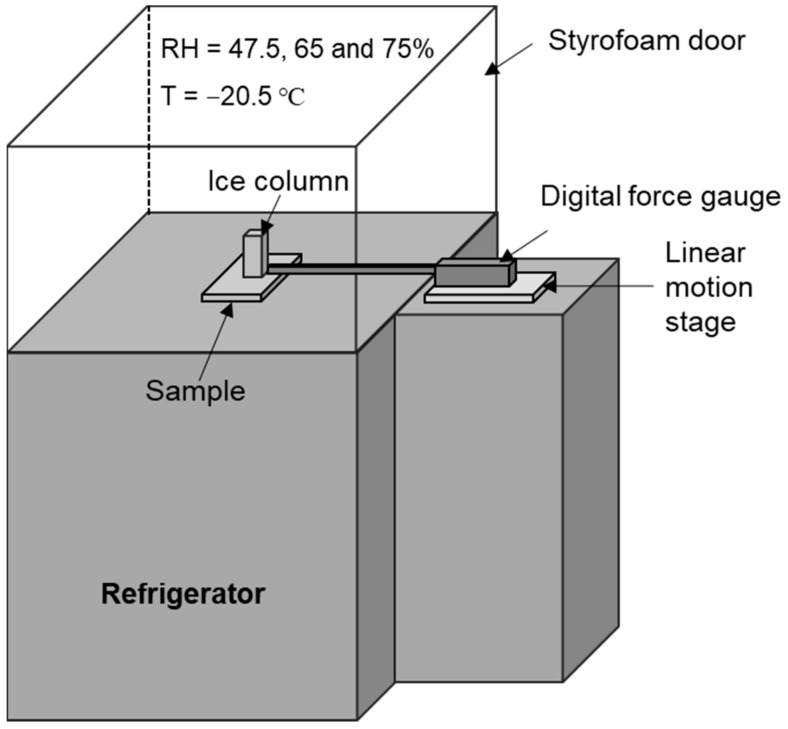
Illustration of a chilling chamber for measuring ice-adhesion strength.

The IAS was measured using a self-made chilling chamber for IAS measurement, as illustrated in Figure 1, with a method described in our previous study [21]. In this technique, a bottomless cuvette (1 cm × 1 cm × 3 cm) was located on a coated surface and contained with 1 mL of deionized (DI) water. By placing the cuvette in a refrigerator, the water column inside was entirely frozen at −20 °C for 3 h under 70% RH. A force transducer (Imada, model ZP, 11) with a 0.8 diameter probe was used to propel the probe perpendicular to the ice column at a predetermined speed in order to quantify the force necessary to separate it from the surface. To reduce torque on the ice column, the probe was positioned less than 2 mm above the substrate. By dividing the maximum force at break by the contact area (1 cm^2^) of the ice–substrate interface, the IAS was determined. Repeat icing and melting tests were carried out by fabricating samples in the same way as for the IAS tests. To create a high/low-alternating-temperature environment, the samples were put in a refrigerator with a light bulb (120 W) 5 cm below them. Cycle #1 is the process by which an ice column was formed by freezing at −25 °C for 90 min (RH = 35%) and then melting with the light bulb for 15 min (*T* = 35 °C, RH = 5%). The IAS was evaluated using the same force transducer after 40 icing/melting cycles to estimate how long the icephobicity would last. The average values were calculated from at least five repetitions.

The icing-delay test was carried out using Perkin Elmer DSC 4000 (USA) by using a method described in a previous study [22]. In this method, the sample surfaces were precisely trimmed to fit into an Al DSC sample pan; 5 μL of DI water was then placed on the 0 °C surfaces. The pan was maintained for 3 min, which ensured that the temperatures of the surface and the water droplet were the same as the temperature of the DSC cooling stage. The temperature was constantly decreased from 0 to −18 °C at a cooling rate of 10 °C min^−1^ and then kept at −18 °C until the water froze entirely to form ice. According to the exothermic peak in the DSC curve, the freezing delay time is the interval between the water droplet making contact with the surface (at 0 °C) and the start of freezing. The freezing delay time values were estimated from the average of five measurements conducted at five positions on each surface.

## 3. Results and Discussion

### 3.1. Fabrication of Hierarchical Superhydrophobic Al Surfaces

**Figure 2:**
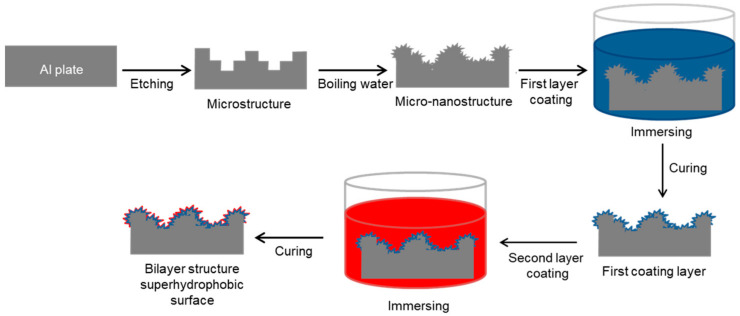
Illustration for the preparation of hierarchically rough Al substrates and superhydrophobic surfaces by sequentially coating low-surface-tension materials.

The general approach for fabricating the hierarchically structured superhydrophobic surfaces on Al plates is illustrated in Figure 2. The Al plates were chemically etched in HCl solution to create a microblock structure. Subsequently, the microstructured Al surface was immersed in boiling water to form nano-leaves on the microblocks. The hierarchical structure was then placed in the first coating solution (PDMS-TES or FD-TMS solution) and dried to generate the first coating layer on the hierarchically rough Al substrate. The sample was soaked in a second coating solution, followed by drying to generate a double-layer coating surface.

**Figure 3:**
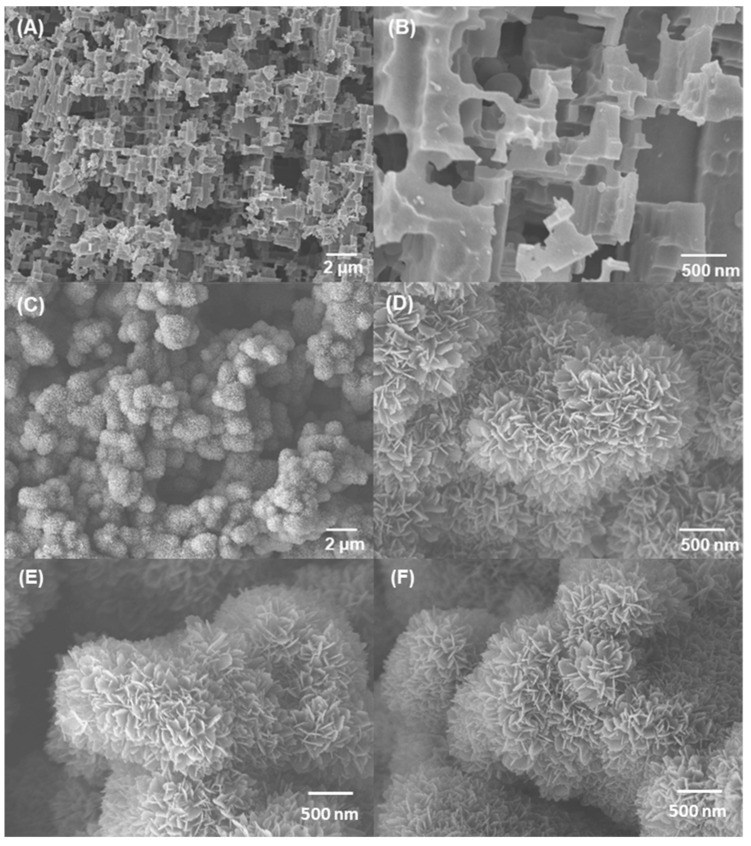
SEM images of the rough Al substrates fabricated by (**A**,**B**) etching in acid solution and by (**C**,**D**) additional treatment in boiling water. SEM images of the (**E**) FD-PDMS_1.92_ and (**F**) PDMS_4.0_-FD coating surfaces.

Figure 3 displays the SEM images of the rough Al substrates. After etching the Al plate in acid solution, microscale patterns (0.2–1.0 μm) with protrusions and pores were formed on the surface (Figure 3A,B). As pointed out in previous literature [23], the formation of a microstructured surface is attributed to the selective corrosion of vulnerable dislocations on the surface of the Al substrate. Subsequently, well-distributed nanostructures were introduced into the microstructure by immersing the micropatterned Al plate in boiling water. Boiling water and the Al surface react chemically to produce boehmite (AlO(OH)) crystals. All microstructures were covered with flower-like nanostructures with thicknesses and lengths of approximately 20 nm and 200 nm, respectively (Figure 3C,D). The hierarchical micro/nanostructured surfaces were then treated with FD-TMS/PDMS-TES solutions, as indicated in the Section 2.

Superhydrophobic surfaces with hierarchical structures (Figure 3E,F) were prepared by sequentially grafting FD-TMS and PDMS-TES in different sequences onto a rough Al plate; these surfaces were compared with the monolayer-coated surfaces with FD or PDMS. During the grafting process, the first deposited material reacts with hydroxyl groups on the Al surface to form a primary coating (or inner coating layer) and is then covered by a secondary coating layer (or outer coating layer) via a further condensation reaction. The difference in reactivity between PDMS-TES and FD-TMS with the Al plate surface, as well as the surface chemistry, result in differences in the liquid repellency and icephobicity of the coated surfaces depending on the coating order. The wetting characteristics of the coated surfaces created by altering the coating-layer sequence are listed in Table 1. The weight percentages of 1H,1H,2H,2H-heptadecafluorodecylsilyl (FD-Si) and polydimethylsiloxanesilyl (PDMS-Si) groups tethered on the rough Al substrate were confirmed by ^1^H NMR analysis. The PDMS-Si ratio was determined by comparing the integrals of the ^1^H NMR signals at 2.15 ppm (-CF_2_-CF_2_-C*H*_2_-) and 0.10 ppm (-O-Si-(C*H*_3_)_2_-), which are associated with the FD-Si and PDMS-Si moieties, respectively. In both double-layer coatings (FD-PDMS_1.92_ and PDMS_4.0_-FD), the weight ratio of PDMS covalently bonded on the Al substrate was much lower than that of the FD moiety. This is ascribed to the steric hindrance and low reactivity of PDMS-TES [24] Compared with the FD-PDMS_1.92_ coating, the content of anchored PDMS on the PDMS_4.0_-FD coating increased. This can be explained by the abundant surface hydroxyl groups of the rough Al surface, which promoted the interaction between PDMS-TES and the surface when PDMS-TES was used in the primary coating step. In contrast, when FD-TMS was used as the primary coating material, in addition to reduction of the surface –OH groups, the low surface tension of the inner FD layer interfered with the access to and condensation of PDMS-TES molecules on the Al surface. For this reason, the ratio of anchored PDMS on the FD-PDMS_1.92_-treated surface was lower than that on the surface treated with PDMS_4.0_-FD.

The superhydrophobic coatings deposited via different sequences significantly affected the wettability of the Al surface, as confirmed by monitoring the CA and SA (SA is correlated with contact angle hysteresis) [25] of water/hexadecane (see Table 1). All samples exhibited exceptional superhydrophobicity with water Cas above 165°. The increased content of PDMS tethered onto the Al surfaces slightly decreased the water CA and increased the SA owing to the comparatively higher surface tension of PDMS than that of FD. Consequently, the PDMS_100_-coated Al surface showed the lowest superhydrophobicity of the four coated surfaces, having a water CA of 165°, which is 10° lower than that of FD_100_, and the SA was 3.8° higher. Along with the surface tension, the sequence of the coating layers, which is related to the surface coverage and surface chemistry, also has a considerable influence on the liquid repellency. When FD-TMS was used in the first step of the coating process, the samples exhibited oleophobic properties, with a hexadecane CA of 150° and 140° for FD_100_ and FD-PDMS_1.92_, respectively. However, the hexadecane CA of FD-PDMS_1.92_ decreased gradually from 140° to 50° within 5 min because of the oleophilicity of the anchored PDMS layer at the solid–liquid interface. In contrast, when PDMS-TES was applied in the initial coating step, highly oleophilic surfaces were obtained, resulting in complete wetting with hexadecane. Notably, although PDMS_4.0_-FD has an extremely low surface tension owing to the outer FD layer, the hexadecane CA approached zero. Because the steric hindrance and the low reactivity of PDMS-TES resulted in low coverage of the Al surface by the primary PDMS layer, ungrafted surface hydroxyl groups remained on the coating surfaces. During the secondary coating with FD-TMS, a condensation reaction occurs between the methoxy functional groups of FD-TMS and the inner PDMS layer, while partially unreacted–OH groups on the surface become pinholes or pores where no coating materials are grafted.

**Figure 4:**
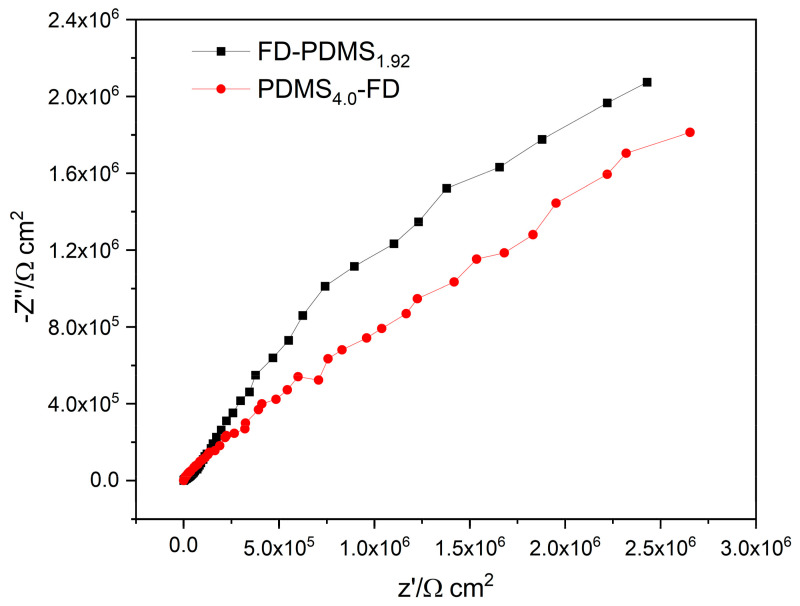
Nyquist plots of Al substrates coated with FD-PDMS_1.92_ and PDMS_4.0_-FD.

The difference in the surface coverage due to the variation of the coating layer sequence on the Al substrate was also verified by XPS and EIS analyses. XPS was used to quantify the elements on the surfaces up to a depth of 10 nm (Table 2). The amount of Al in the sample was affected by the coating thickness and surface coverage. The Al concentration of the Al surface coated with FD-PDMS_1.92_ was 17.70%, which is slightly lower than that of the FD_100_ surface, indicating that a thicker coating was formed by FD-PDMS_1.92_. This is attributed to the PDMS chains being longer than the FD chains. In contrast, the Al content of PDMS_4.0_-FD and PDMS_100_ was 18.99% and 20.80%, respectively, indicating incomplete coverage of the Al surfaces by the coating materials. In other words, when PDMS was used as the initial coating material, the low chemically reactive TES group and the bulky PDMS chains resulted in non-uniform or incomplete surface coverage. Adding a small fraction of PDMS as a secondary coating layer can enhance the superhydrophobicity and surface coverage of the coating materials. The defect structure and surface coverage of the FD-PDMS_1.92_ and PDMS_4.0_-FD surfaces, which influence the wettability and icephobicity, were further evaluated and compared by EIS in aqueous chloride. EIS is a useful method for determining the quality of coatings by examining their impact on electron-transfer reactions [26,27]. Figure 4 shows the Nyquist plots of the PDMS_4.0_-FD and FD-PDMS_1.92_ surfaces in sodium chloride solution. The different diameters of the capacitive loops are attributed to differences in the charge-transfer resistance of the coated surfaces. FD-PDMS_1.92_ exhibited higher impedance in a 3.5 wt.% NaCl solution, demonstrating that the coating was able to cover more surface area and offer a higher level of resistance to charge transfer.

### 3.2. Icephobic Properties

**Figure 5:**
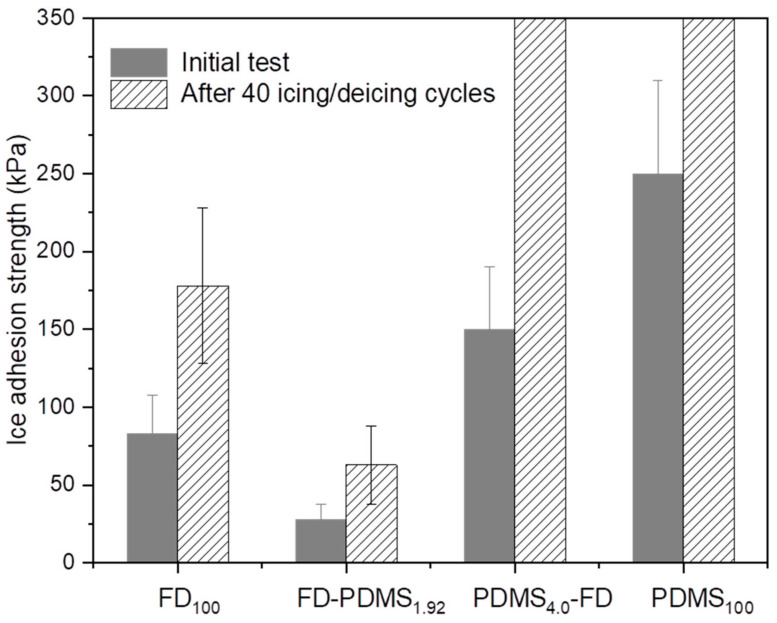
Ice adhesion strengths for rough Al surfaces coated with FD_100_, FD-PDMS_1.92_, PDMS_4.0_-FD, and PDMS_100_ before and after 40 icing/melting cycles. The tests for PDMS_4.0_-FD and PDMS_100_ after 40 cycles failed owing to breakage of the ice columns.

The IAS of the superhydrophobic surfaces was tested to evaluate the icephobic performance [7]. Figure 5 shows the IASs of the Al surfaces coated with FD_100_, FD-PDMS_1.92_, PDMS_4.0_-FD, and PDMS_100_ at −20 °C and 70% RH. The IAS of the FD_100_ surface at the first cycle was 83 kPa owing to the extremely low surface tension of the fluorine components. Remarkably, FD-PDMS_1.92_ exhibited the lowest IAS (28 kPa), which was almost three times lower than that of the PDMS-free sample. This can be explained by the synergistic effects of the low surface tension and dense coverage of FD and the lubricating property of PDMS [17,21]. The use of FD-TMS in the initial coating stage resulted in higher surface coverage and a lower surface tension than those of PDMS-TES. This stabilizes the water droplets in the Cassie–Baxter state, even at low temperature and high RH [28]. Coating PDMS onto the inner FD layer in the secondary coating step also enables interfacial slippage at the ice-coating interface owing to the low *T_g_*, high elasticity, and low interaction of the PDMS moieties with ice/water, which further reduces the IAS [21]. In contrast, when PDMS was introduced onto the surface as an inner coating layer, the ice adhesion on the PDMS_4.0_-FD and the PDMS_100_ increased considerably to 150 kPa and 250 kPa, respectively. Although the double coatings (FD-PDMS_1.92_ and PDMS_4.0_-FD) include both FD with low surface tension and PDMS with lubricating properties, the IAS on FD-PDMS_1.92_ was approximately 5-fold lower than that on the PDMS_4.0_-FD surface. This is attributed to the change in the surface wetting state depending on the grafting sequence of FD and PDMS. Although the PDMS_4.0_-FD had a low surface tension because of the outer FD layer, the poor surface coverage generated from the inner PDMS layer induced a transition in wetting mode from the Cassie–Baxter state to the Wenzel state by the filling cavities with freezing water [8]. Consequently, the IAS on the surface increased owing to the firm anchoring of the ice into the micro-nanostructure.

**Figure 6:**
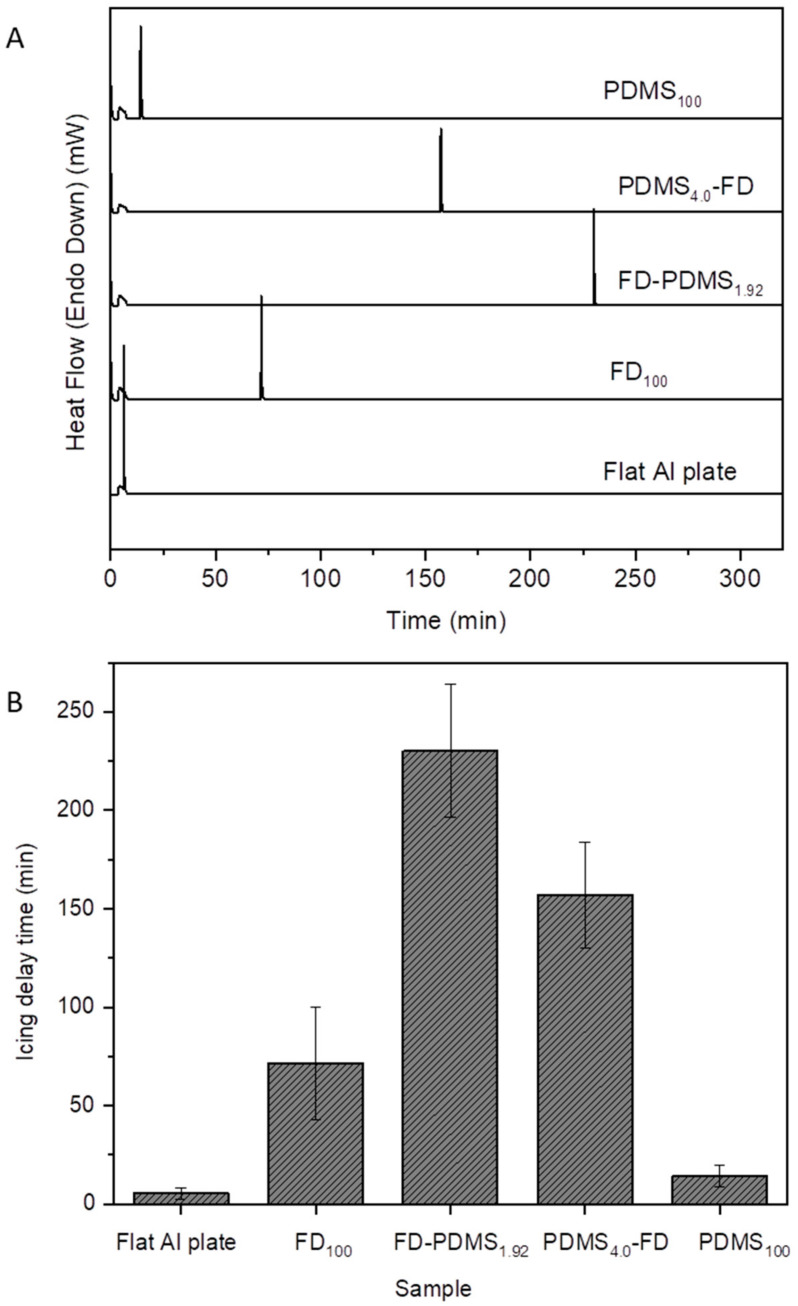
(**A**) DSC graphs of super-cooled water droplets dropped on flat and rough Al surfaces coated with FD_100_, FD-PDMS_1.92_, PDMS_4.0_-FD, and PDMS_100_. The temperature was programmed to drop from 0 to −18 °C at a rate of 10 °C/min^−1^ and was retained at −18 °C. (**B**) Icing delay times of the surfaces.

The grafting sequence changes the wettability state and the interaction between the ice and the coated surface. This has a significant impact on how long water takes to freeze on coated surfaces. Figure 6A,B shows the freezing delay times of water droplets on the superhydrophobic Al surfaces and the flat Al plate. Compared with the water droplets on the untreated Al surface, the droplets on the superhydrophobic coatings exhibited a relatively longer freezing delay time. Notably, ice was already formed at −15.3 °C on the flat Al surface before reaching the measurement temperature of −18 °C, whereas the freezing delay time was 71.5 min for FD_100_. FD, with hierarchical surface roughness and low surface tension, significantly contributed to entrapping a large number of air pockets and reducing the contact area between the water droplet and the substrate surface, resulting in substantially reduced heat transfer efficiency and delayed ice formation on the surface. The double coatings (PDMS_4.0_-FD and FD-PDMS_1.92_) exhibited much longer freezing delay times of 157 and 230 min, respectively, compared to FD_100_ and the flat Al plate. Due to the comparatively dense FD coating and the low interaction between the PDMS outer coating and the water, the water droplet exhibited the longest freezing delay time on FD-PDMS_1.92_. The insulating nature of these substrates is primarily responsible for the previously documented delayed freezing of water on superhydrophobic substrates [13,29]. The high density and low surface tension of the FD layer reduce the heat transfer length, while the weak interaction between ice (or water) and the outer PDMS layer depresses the ice–surface contact area. Consequently, there is a lower chance of heterogeneous nucleation at the water–surface interface and a higher free-energy barrier to the formation of a significant nucleus [30]. Mutual synergy is operative between the components of PDMS_4.0_-FD, but the relatively lower coating density decreased the freezing delay time. Therefore, among the four coated surfaces, the shortest icing delay time was obtained with PDMS_100_. In other words, the low surface coverage owing to the initial PDMS layer promoted heat transfer through the incompletely uncovered Al surface, speeding up ice production. Moreover, the condensation of water vapor on the uncovered Al surface reinforced the adhesion of ice to the Al substrate.

### 3.3. Durability of the Icephobic Surface

**Figure 7:**
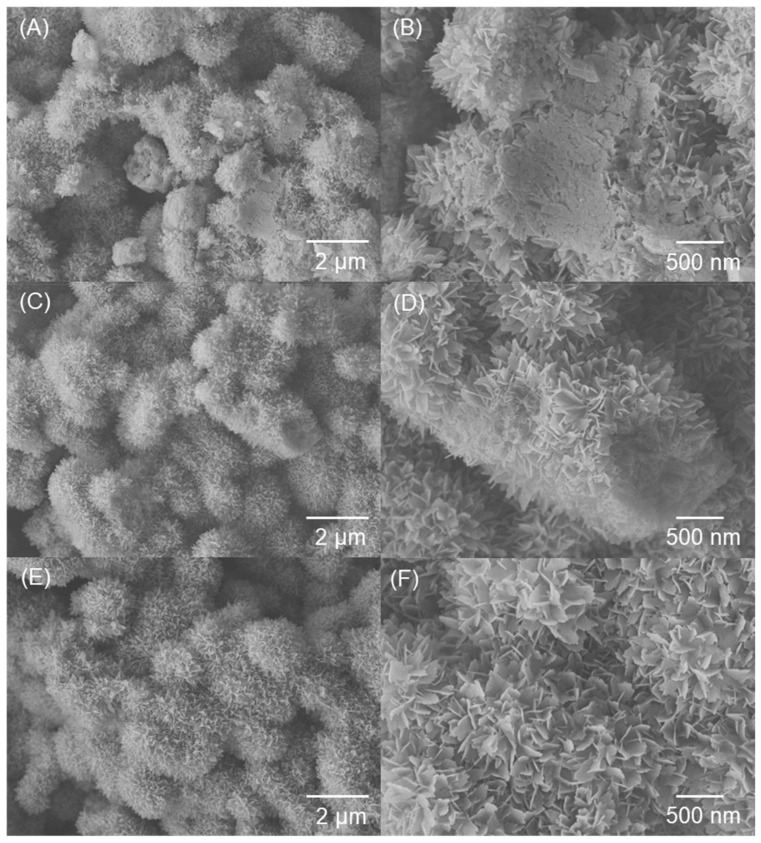
SEM images of (**A**,**B**) PDMS_100_, (**C**,**D**) FD_100_, and (**E**,**F**) FD-PDMS_1.92_ after 40 icing/melting cycles.

Generally, superhydrophobic surfaces often have low IAS and delayed nucleation of water molecules to form surface ice, but the durability of the surfaces is of primary concern. Superhydrophobic surfaces suffer from weak mechanical durability owing to the fragility of their nano/micro-surface structures. The hierarchical structure is harmed during the freezing process as a result of the water expanding and pressing up against the substrate, which raises the ice-adhesion strength and lowers durability. Therefore, the construction of superhydrophobic surfaces with mechanical durability is necessary to expand their practical applications in the real world. By measuring the IASs of the coated Al surfaces after 40 icing/melting cycles, the durability of the superhydrophobic surfaces was examined in this study (Figure 5). The hydrophobicity decreased and the IAS increased for all samples after 40 icing/melting cycles, owing to damage of the surface structure during icing/melting. After 40 icing/melting cycles, the ice column was broken, and ice residue was still present on the PDMS_4.0_-FD- and PDMS_100_-coated Al surfaces. This is indicative of cohesive failure of ice on the surfaces. The damage to the hierarchical surfaces and the eventual loss of their ice-releasing properties after multiple icing/melting cycles indicate the large contribution of the mechanical interlocking effect on the surfaces owing to the low surface coverage of the coatings. The FD_100_ coating exhibited improved durability because of its high density and low surface tension; thus, the IAS evaluated after 40 cycles was 178 kPa. Notably, after 40 icing/melting cycles, the FD-PDMS_1.92_-coated surface exhibited the lowest ice-adhesion strength, which was 63 kPa. This value is much lower than that of FD_100_ obtained in the first cycle (83 kPa), and notably lesser when compared with previous reports [15,31,32]. Along with having the effect of depressing the initial ice adhesion, PDMS also has the ability to reduce damage to the topological nano/microstructure throughout the icing/melting tests, which is connected with the low ice-adhesion strength. Figure 7 shows the damaged surface area of FD-PDMS_1.92_ compared with that of FD_100_ and PDMS_100_ after 40 icing/melting cycles. The nanoscale structures of the FD_100_ and PDMS_100_ surfaces were severely damaged after the durability test, resulting in increased surface tension and a larger ice–surface contact area. Consequently, water easily penetrated the rough grooves, leading to physical interlocking between the ice and the substrate and resulting in stronger adhesion. After the durability test, no significant surface degradation was seen on FD-PDMS_1.92_, proving the positive effects of these materials in achieving durable icephobicity. Therefore, we anticipate that the manufacturing process for anti-icing surfaces described here will satisfy the demands of real-world applications.

## 4. Conclusions

Superhydrophobic surfaces with high anti-icing properties and robustness were designed and fabricated by sequentially coating hierarchical nano-microstructured Al substrates with FD and PDMS. By changing the coating sequences, it was possible to achieve superior icephobicity, which corresponds to a lower IAS and prolonged freezing time. The FD-PDMS1.92 coating with an inner FD layer and outer PDMS layer exhibited the lowest IAS of 28 kPa (at −20 °C and 70% RH) and the longest freezing delay time of 230 min (at −18 °C). These features are attributed to the incorporation of a dense coating layer with a low-surface-tension fluorinated component and the high mobility of PDMS. The introduction of PDMS onto the coating surface plays a vital role in weakening the ice–surface interaction and enhancing the durability of icephobicity. FD-PDMS1.92 showed a low IAS of 63 kPa after 40 icing/melting cycles, which was much lower than that of FD100 obtained in the first cycle (83 kPa).

## Figures and Tables

**Table 1:** PDMS contents of the coated materials and wettability parameters of the superhydrophobic surfaces.

**Sample**	**Incorporated PDMS Content (wt.%) ^1^**	**Water** **Contact Angle (°)**	**Water Sliding Angle (°)**	**Hexadecane Contact Angle (°)**
FD_100_	0	176.5 ± 1.5	1.4 ± 0.2	150
FD-PDMS_1.92_	1.92	173.0 ± 1.5	2.2 ± 0.5	140→50
PDMS_4.0_-FD	4.0	170.5 ± 1.0	4.8 ± 0.8	0
PDMS_100_	100	165.0 ± 0.5	5.2 ± 1.2	0

^1^ Calculated from the ^1^H NMR data.

**Table 2:** Elemental compositions of the coatings on the rough Al substrates.

**Atom**	**XPS Atomic Concentration (%)**			
	**FD_100_**	**FD-PDMS_1.92_**	**PDMS_4.0_-FD**	**PDMS_100_**
Si 2p	3.61	3.88	6.71	10.67
C 1s	15.12	18.28	23.19	23.21
Al 2p	18.62	17.70	18.99	20.80
O 1s	34.69	33.57	40.91	45.32
F 1s	27.97	26.58	10.2	0

## Data Availability

Not applicable.
